# Low cytotoxicity of linoleic acid-derived epoxy-fatty acids in liver and colon cell lines

**DOI:** 10.1016/j.bbrep.2026.102607

**Published:** 2026-04-28

**Authors:** Henrik Reuter, Nadja Kampschulte, Kathrin Plitzko, Katja Mosel, Christophe Morisseau, Nils Helge Schebb

**Affiliations:** a Food Chemistry, School of Mathematics and Natural Sciences, University of Wuppertal, Gaussstrasse 20, Wuppertal, 42119, Germany; b Department of Entomology and Nematology, and UC Davis Comprehensive Cancer Center, University of California Davis, Davis, CA, 95616, USA

**Keywords:** Oxidized fats, Oxidized oils, HepG2 cells, Caco2 cells, EpOME, Oxylipins

## Abstract

Linoleic acid-derived epoxy-fatty acids (EpOMEs) are found in oxidized plant oils and processed foods. Consumption of oxidized oils may lead to a variety of negative health effects. Due to the assumed chemical reactivity of the epoxy-group, toxicity of epoxy-fatty acids, such as 9(10)-EpOME and 12(13)-EpOME, is frequently discussed. Therefore, we investigated the effect of 9(10)-EpOME and 12(13)-EpOME on cell viability by evaluating cell proliferation, LDH leakage, lysosomal integrity and dehydrogenase activity in HepG2 and Caco2 cells. Both tested epoxy-fatty acids show no or low cytotoxicity only above 50 μM in both cell lines, with 12(13)-EpOME being slightly more cytotoxic. This concentration is well above endogenous levels of EpOMEs in circulation.

## Highlights

• Investigation of cytotoxic effects of 9(10)-EpOME and 12(13)-EpOME.
• Both tested epoxy-fatty acids show no or low cytotoxicity only above 50 μM in HepG2 and Caco2 cells.
• 12(13)-EpOME was slightly more cytotoxic.
• Toxic concentrations are below endogenous levels of EpOMEs in circulation.

## 3 Abbreviations

**DiHOME**: dihydroxy-octadecenoic acid
**EKODE**: 9-oxo-11-(3-pentyloxiran-2-yl)undec-10-enoic acid
**EpOME**: epoxy-octadecenoic acid
**FBS**: fetal bovine serum
**LA**: linoleic acid
**LC-ESI(−)-MS/MS**: liquid chromatography with negative electrospray ionization coupled tandem-mass spectrometry
**LDH**: lactate dehydrogenase
***m*CPBA**: *meta*-chloroperoxybenzoic acid
**PUFA**: polyunsaturated fatty acid

## 1 Introduction

Vegetable oils are important foods in the Western diet [1]. Plant oils contain oxidized fatty acids, including epoxy-polyunsaturated fatty acids (PUFAs) and dihydroxy-PUFAs [2]. Particularly, frying and deep frying processes lead to increased formation of oxidized PUFAs, i.e., oxylipins [[3], [4], [5]]. Epidemiological studies suggest that the consumption of oxidized edible oils may lead to negative health effects, including cancer in various areas of the gastrointestinal tract and chronic heart disease [6,7]. In a recent study, consumption of oxidized corn oil led to an increase in the severity of colitis, changed the composition of gut bacteria and increased the occurrence of colorectal cancer in mice [8].

Epoxy-PUFAs of all biologically occurring PUFAs are found in biological samples. Particularly, arachidonic acid, eicosapentaenoic acid and docosahexaenoic acid-derived epoxy-PUFAs are lipid mediators regulating inflammation, blood pressure, angiogenesis and tumorigenesis without toxic effects [9,10]. The biological effects of linoleic acid-derived EpOMEs and their hydrolysis products (DiHOMEs) are summarized in a recent review [11]. The chemical reactivity of the epoxide-group, shown for a variety of epoxy-compounds [12,13], led to the hypothesis that epoxy-PUFAs could react with cellular (macro)molecules, which might contribute to the negative health effects reported for oxidized oils. In two independent studies, concentrations from 34 to 2700 nmol/g and 23 to 930 nmol/g of the LA-derived oxylipins 9(10)-epoxy octadecenoic acid (EpOME) and 12(13)-EpOME were found in edible oils [14] and margarine [15]. In processed foods, levels of 5 to 30 nmol/g and 2.5 to 300 nmol/g of 9(10)-EpOME and 12(13)-EpOME were found, respectively [14]. Epoxy-PUFAs and epoxy-triacylglyceroles are found to be readily absorbed following oral intake by human subjects [16,17]. The genotoxic effects of oxidized edible oils tested in an artificial system suggest epoxy-PUFAs as the possible cause [18,19], whereas other groups did not find genotoxic effects of specific epoxy-PUFAs [20]. The epoxide of oleic acid, epoxy-stearic acid, was shown to decrease cell viability in a dose- and time-dependent manner in HepG2 cells at concentrations in the cultivation medium from 100 to 500 μM [21,22].

Currently, data for direct cytotoxicity of LA-derived epoxy-PUFAs is scarce and inconsistent. Cytotoxic effects of epoxy-PUFAs, including LA-derived 9(10)-EpOME and 12(13)-EpOME, were shown to depend on the expression of soluble epoxide hydrolase in an insect cell line [23,24] occurring at concentrations of 150 μM and higher. In order to assess the direct toxic effects of LA-derived epoxy-PUFAs, the cytotoxicity of 9(10)-EpOME and 12(13)-EpOME was investigated in this study using several endpoints of cell viability in HepG2 and Caco2 cells. The supplementation was monitored using liquid chromatography with negative electrospray ionization coupled tandem-mass spectrometry (LC-ESI(−)-MS/MS).

## 2 Materials and methods

### 2.1 Materials

HepG2 human liver carcinoma cells were purchased from the German Collection of Microorganisms and Cell Cultures GmbH (DSMZ, Braunschweig, Germany). Caco2 human colorectal adenocarcinoma cells were purchased from the American Type Culture Collection (ATCC, local distributor LGC Standards GmbH, Wesel, Germany).

LA and minimum essential medium were supplied by Thermo Scientific (Schwerte, Germany). Non-essential amino acid solution, penicillin-streptomycin solution, l-Gln solution and *meta*-chloroperoxybenzoic acid (*m*CPBA) were supplied by Sigma-Aldrich (Taufkirchen, Germany). Fetal bovine serum (FBS) (superior standardized) was supplied by Biochrom (Berlin, Germany). The soluble epoxide hydrolase inhibitors 2213, *t*-TUCB and TPPU were kindly donated by the laboratory of Professor Bruce Hammock at U.C Davis, CA. All other standard chemicals and solvents were supplied by Merck (Darmstadt, Germany).

The LA-derived epoxy-PUFAs 9(10)-EpOME and 12(13)-EpOME were synthesized from LA-ethyl ester using *m*CPBA, yielding two regioisomers [25,26]. After separation by column chromatography, both regioisomers were saponified as described before [25]. Identity and purity were controlled by nuclear magnetic resonance spectroscopy and LC-ESI(−)-MS/MS analysis [[27], [28], [29], [30]]. Stock solutions of epoxy-PUFAs were prepared in ethanol and diluted with cell culture medium to final concentrations for cytotoxicity assays. Concentrations of supplemented oxylipins and corresponding dihydroxy-FAs in the blank medium, and the supplemented levels were monitored by targeted LC-ESI(−)-MS/MS analysis [[27], [28], [29], [30]] (Supplemental Material, Fig. S1). Statistical analysis was performed with GraphPad Prism (GraphPad Software, Boston, MA, USA).

### 2.2 Cell culture

Cells were grown in Eagle's Minimum Essential Medium supplemented with 10% FBS, 100 μM non-essential amino acids (l-Ala, l-Asn, l-Asp, l-Glu, Gly, l-Pro, l-Ser), 2 mM l-Gln and 100 U/mL penicillin and 100 μg/mL streptomycin in a humidified incubator at 37 °C and 5% CO_2_ atmosphere on 60.1 cm^2^ cell culture dishes at a density of 250.000 cells/mL and 100.000 cells/mL for HepG2 and Caco2, respectively, with 10 mL medium per dish. Assays were carried out on well plates with the same density, with 375.000 cells and 150.000 cells per well on 6-well plates, 75.000 cells and 30.000 cells per well on 24-well plates and 15.000 cells and 6000 cells per well on 96-well plates for HepG2 and Caco2, respectively.

### 2.3 Assessment of cell proliferation and cytotoxicity

Cell proliferation as a marker for cell viability was carried out as described before [31]. After adhesion, medium was removed and cells were incubated with medium containing epoxy-PUFAs at 5 to 100 μM (n = 3) for 24 h to give cells enough time to complete a full cell cycle including proliferation. Cells were harvested by scraping and viable cells were counted by CASY Cell Counter and Analyzer (OMNI Life Science, Bremen, Germany). Cell counts of medium controls were defined as 100%.

Outer cell membrane damage was assessed by lactatde hydrogenase (LDH) leakage assay as described before [31]. After adhesion, medium was removed and cells were incubated with medium containing epoxy-PUFAs at 10 to 130 μM or Triton X 0.2% as a positive control (n = 4) for 2 or 4 h. To assess acute cell membrane damage, only short incubation times are evaluated because the activity of the released LDH decreases over longer incubation times. The sum of intra- and extracellular LDH activity was calculated, and extracellular LDH activity of samples was compared to the release from vehicle controls (0.1% ethanol *v*/*v*), which was defined as 0% release.

Lysosomal integrity as a marker for cell membrane damage was assessed by neutral red uptake assay as described before [32]. After adhesion, medium was removed and cells were incubated with medium containing epoxy-PUFAs at 10 to 100 μM or sodium dodecyl sulfate 0.1% as a positive control (n = 6) for 2, 4 or 24 h to assess short- and long-term effects on lysosomal integrity. Absorption of medium controls was defined as 100% lysosomal integrity.

Dehydrogenase activity, as a marker for cellular metabolic activity, was assessed by AlamarBlue assay as described before [33]. After adhesion, medium was removed and cells were incubated with medium containing epoxy-PUFAs at 10 to 130 μM or staurosporin 20 μM as a positive control (n = 6) for 2, 4 or 24 h to assess short- and long-term effects on dehydrogenase activity. Fluorescence of medium controls was defined as 100% metabolic activity.

### 2.4 Quantitative targeted LC-ESI(−)-MS/MS analysis of oxylipins

Quantitative targeted LC-ESI(−)-MS/MS analysis of oxylipins was carried out using a 1290 Infinity II LC system (Agilent Technologies, Waldbronn, Germany) coupled to a QTRAP 5500 mass spectrometer (Sciex, Darmstadt, Germany) as described before [[27], [28], [29], [30]]. Chromatographic separation was carried out on a C18 reversed-phase column (Zorbax Eclipse Plus C18, 2.1 × 150 mm, particle size 1.8 μm, pore size 9.5 nm, Agilent Technologies) equipped with a guard column (SecurityGuard Ultra C18 cartridge, 2.1 × 2 mm, Phenomenex, Aschaffenburg, Germany) at 40 °C with 0.1% acetic acid in H_2_O containing eluent B (95:5, *v*/*v;* eluent A) and a mixture of acetonitrile, methanol and acetic acid (800:150:1, *v*/*v*/*v;* eluent B) at a flow rate of 300 μL/min. The injection volume was set to 5 μL. Oxylipins were detected in scheduled selected reaction monitoring mode following negative electrospray ionization and quantified using external calibration with isotopically labeled oxylipins as internal standards [27,28].

## 3 Results and discussion

**Fig. 1:**
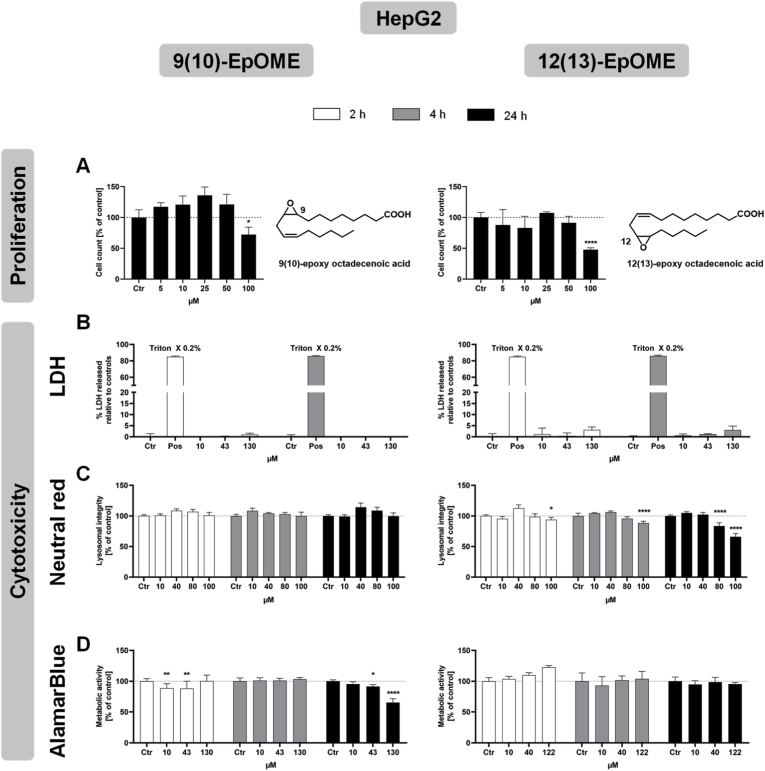
**Cytotoxicity of epoxidized linoleic acid in HepG2 cells.** Cells were incubated with 9(10)-EpOME or 12(13)-EpOME for 2, 4 and 24 h (white, grey and black bars), and cell proliferation over 24 h (A) as well as cytotoxicity by LDH release assay (B), neutral red uptake assay (C) and AlamarBlue assay (D) were assessed. All data are shown as mean +SD (n = 3-6) (Two-way ANOVA followed by Dunnett's multiple comparisons test, ∗p < 0.05; ∗∗p < 0.01; ∗∗∗p < 0.001; ∗∗∗∗p < 0.0001, compared to control), with Ctr: vehicle control for LDH release assay and medium control for proliferation assay, neutral red assay and AlamarBlue assay, and Pos: positive control.

**Fig. 2:**
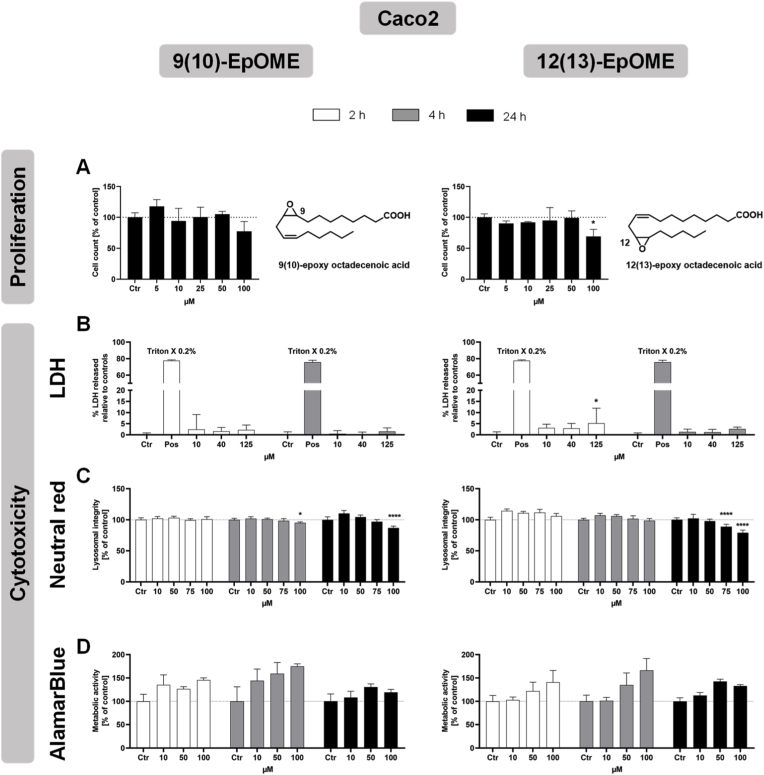
**Cytotoxicity of epoxidized linoleic acid in Caco2 cells.** Cells were incubated with 9(10)-EpOME or 12(13)-EpOME for 2, 4 and 24 h (white, grey and black bars), and cell proliferation over 24 h (A) as well as cytotoxicity by LDH release assay (B), neutral red uptake assay (C) and AlamarBlue assay (D) were assessed. All data are shown as mean +SD (n = 3-6) (Two-way ANOVA followed by Dunnett's multiple comparisons test, ∗p < 0.05; ∗∗p < 0.01; ∗∗∗p < 0.001; ∗∗∗∗p < 0.0001, compared to control), with Ctr: vehicle control for LDH release assay and medium control for proliferation assay, neutral red assay and AlamarBlue assay, and Pos: positive control.

The effect on cell viability is an important first step in assessing the potential toxicity of presumably reactive epoxy-PUFAs. We investigated the effects of 9(10)-EpOME and 12(13)-EpOME on cell proliferation, and three different cytotoxicity endpoints in HepG2 (Fig. 1) and CaCo2 (Fig. 2) cell lines. The concentration in the cell culture medium was controlled by LC-ESI(−)-MS/MS [[28], [29], [30]]. The actual concentration of the epoxy-PUFA in the medium was in the expected concentration range and the solution was stable (Supplemental Material, Figs. S1–S2). Assays were carried out up to the highest soluble concentration in cell culture medium with 0.1% ethanol (*v*/*v*) at about 130 μM. The exact concentration in the media was controlled by LC-MS and differed slightly from the added amount, possibly due to the handling of small volumes of EtOH and absorption to the plastic material.

Cell proliferation was decreased above 50 μM with both tested epoxy-fatty acids in both cell types, with a slightly stronger effect from 12(13)-EpOME (Figs. 1A and 2A).

There was no clear indication of a biologically relevant release of LDH (<10% relative to controls), neither for increased concentrations nor for longer incubation times of up to 4 h (Figs. 1B and 2B), indicating no acute plasma membrane damage by both oxylipins up to a concentration of 130 μM.

In the neutral red assay, no effect on lysosomal integrity was observed for up to 100 μM 9(10)-EpOME in HepG2 cells and a slight decrease above 75 μM 9(10)-EpOME in Caco2 cells after 24 h incubation (Figs. 1C and 2C). Incubation with 12(13)-EpOME for 24 h led to a decrease in lysosomal integrity above 50 μM in both cell lines (Figs. 1C and 2C). This shows that both cell lines tolerate higher levels of 9(10)-EpOME than of 12(13)-EpOME, indicating that 12(13)-EpOME has higher cytotoxic potential regarding lysosomal membrane integrity.

In the AlamarBlue assay, a significant decrease in metabolic activity was only observed in HepG2 cells incubated with 9(10)-EpOME for 24 h at concentrations of 43 μM or higher. No decrease was observed for 12(13)-EpOME (Fig. 1D). No significant decrease of metabolic activity was observed in Caco2 for both epoxy-fatty acids (Fig. 2D). This leads to the conclusion that metabolic activity might be cell type specifically impaired by high concentrations of 9(10)-EpOME.

Previous studies showed also a low cytotoxicity of epoxy-fatty acids in HepG2 cells. Liu et al. [21] found a time- and dose-dependent decrease in metabolic activity by epoxy-stearic acid, at 10 μM, while Kitaguchi et al. [20] found an effect on the metabolic activity at 250 μM and an LDH release at concentrations above 100 μM. Moghaddam et al. [23] reported a decrease in metabolic activity at concentrations from 90 to 370 μM of epoxy-PUFA ethyl esters in Sf-21 cells. However, it should be noted that the concentrations of the nonesterified epoxy-PUFAs above 150 μM investigated in these studies led in our hands to a precipitation of the compounds in the water-based cell culture medium.

Greene et al. [24] showed that the corresponding dihydroxy-LAs, i.e., 9,10-DiHOME and 12,13-DiHOME are more toxic than the EpOMEs. In our study, following the incubation with EpOMEs, only minor amounts of DiHOMEs were formed (Supplemental Material, Figs. S1–S2). Moreover, co-incubation with the potent soluble epoxide hydrolase inhibitor TPPU and two structurally diverse inhibitors [[34], [35], [36]], which prevent the hydrolysis of EpOMEs to DiHOMEs, led to an increase of the toxicity of EpOMEs (Supplemental Material, Figs. S3–S4). Thus, the observed effects on cell viability in our studies seem to result from the epoxy-PUFAs and not from their hydrolysis products.

Generally consistent with previous studies on epoxy-stearic acid [21,22], our study also showed that the LA-derived epoxy-PUFAs 9(10)-EpOME and 12(13)-EpOME exhibited weak cytotoxicity at concentrations above 50 μM. These findings are supported by the reduced cell proliferation occurring at 100 μM in both HepG2 and Caco2 cell lines.

## 4 Conclusions

Both LA-derived epoxy-PUFAs show low cytotoxicity in human cell lines derived from liver and from colon. Only concentrations above 50 μM reduced the proliferation of the cell lines and generated low cytotoxic effects on metabolic activity and lysosomal integrity, with 12(13)-EpOME being slightly more toxic based on the assessment of lysosomal integrity. It should be noted that concentrations above 150 μM of the lipophilic fatty acid derivatives are not soluble in cell culture medium and thus cannot be tested. Nevertheless, one cannot rule out potential mutagenic or genotoxic effects. However, at the high concentrations tested, a strong genotoxic agent should have also caused reduced cell proliferation. Thus, different from other epoxide containing molecules, e.g. reactive metabolites of acrylamide and polycyclic aromatic hydrocarbon, epoxy-PUFAs seem to have a low toxicity due to their low chemical reactivity.

Regarding possible exposure and health effects, one should take into account that both tested epoxy-PUFAs occur at high concentrations in food [14,15]. The found concentrations of 9(10)-EpOME and 12(13)-EpOME in vegetable oils are in the same range as the concentrations that showed cytotoxicity. However, the actual exposure of liver or colon cells following ingestion of epoxy-PUFAs bound in triacylglycerols cannot be easily determined and it is impossible to directly extrapolate possible health effects from cell culture data. Moreover, the cell lines used for the cytotoxicity assays were carried out in cancer-derived cells and may not fully reflect the physiological responses of normal human liver and intestinal tissues. These cancerous cell lines are known to exhibit altered metabolic activity, redox balance, proliferation rates, and lipid handling compared to non-cancerous cell lines or primary cells, which could influence both the uptake and intracellular processing of EpOMEs. Consequently, the observed low cytotoxicity of EpOMEs may not accurately reflect their effects in non-cancerous cells, where metabolic activity, membrane composition, and signaling responses may differ. Therefore, further studies using more physiologically relevant models, including non-cancerous cell lines, primary human cells, precision-cut tissue slices, or animal models, are warranted to better extrapolate the observed effects of EpOMEs to human health.

Negative health effects from oxidized edible oils may also result from effects not associated with direct cellular damage by epoxy-PUFAs but rather by other oxylipins. Wang et al. [8] showed that consumption of oxidized oils promotes colitis and changes in the gut microbiome in a mouse model even though the oxidized oils did not contain higher levels of LA-derived epoxy-PUFAs compared to the unoxidized oil, whereas the levels of hydroxy-PUFAs, oxo-PUFAs, trihydroxy-PUFAs and of the highly reactive 9-oxo-11-(3-pentyloxiran-2-yl)undec-10-enoic acid (EKODE) were significantly increased during oxidation. Moreover, it has to be taken into account that enzymatic formation of EpOMEs from LA and its metabolism in the human body is a major factor controlling the levels of EpOMEs in circulation [37], which are around 0.5 μM, while concentrations resulting in some toxicity were shown to be much higher than observed endogenous levels.

## CRediT authorship contribution statement

**Henrik Reuter:** Data curation, Formal analysis, Investigation, Visualization, Writing – original draft, Writing – review & editing. **Nadja Kampschulte:** Investigation, Methodology, Writing – original draft, Writing – review & editing. **Kathrin Plitzko:** Investigation, Methodology, Resources, Writing – review & editing. **Katja Mosel:** Methodology, Resources, Writing – review & editing. **Christophe Morisseau:** Resources, Writing – review & editing. **Nils Helge Schebb:** Conceptualization, Methodology, Supervision, Writing – original draft, Writing – review & editing.

## Declaration of competing interest

The authors declare that they have no known competing financial interests or personal relationships that could have appeared to influence the work reported in this paper.

## Data Availability

Data will be made available on request.
