# Supplementary material for: Low cytotoxicity of linoleic acid-derived epoxy-fatty acids in liver and colon cell lines
Source: Biochem Biophys Rep. 2026 Apr 28;46:102607. doi: 10.1016/j.bbrep.2026.102607 (PMC13139969; doi:10.1016/j.bbrep.2026.102607)
Supplement: Multimedia component 1 [file mmc1.pdf]

Supplemental Material

Low cytotoxicity of linoleic acid-derived epoxy-fatty acids in liver and colon cell lines

Henrik Reuter[1], Nadja Kampschulte[1], Kathrin Plitzko[1], Katja Mosel[1], Christophe Morisseau[2], Nils Helge Schebb[1]*

[1]Food Chemistry, School of Mathematics and Natural Sciences, University of Wuppertal, Gaussstrasse 20, 42119, Wuppertal, Germany

[2]Department of Entomology and Nematology, and UC Davis Comprehensive Cancer Center, University of California Davis, Davis, CA 95616, USA

*corresponding author: Nils Helge Schebb, Food Chemistry, School of Mathematics and Natural Sciences, University of Wuppertal, Gausstrasse 20, 42119 Wuppertal, Germany; Email: nils@schebb-web.de; Phone: +49-202-439-3457

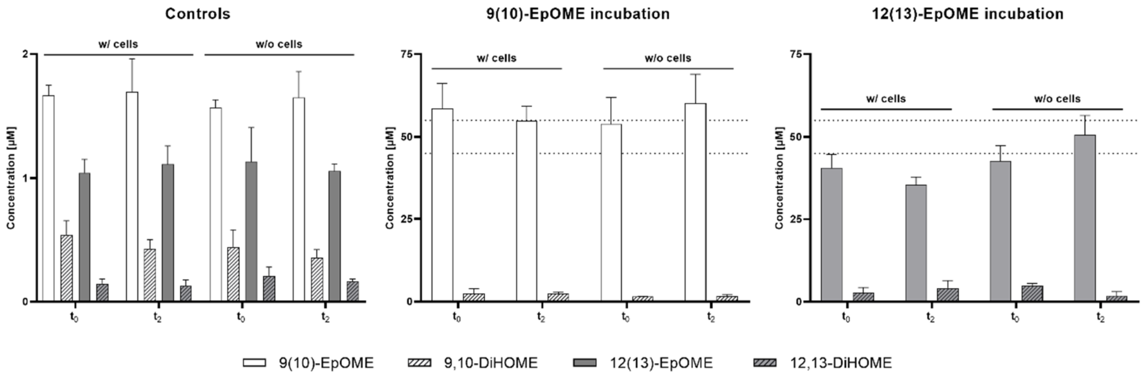

Figure S1: Concentration of epoxy-LA (EpOME) and dihydroxy-LA (DiHOME) in cell culture medium during incubation. Cell culture medium of was analyzed at the start of the incubation with epoxy-LA (t_0) and after 2 h (t_2) in wells seeded with Caco2 cells (w/ cells) and in wells with no cells (w/o cells). LC-ESI(-)-MS/MS determined concentrations are shown exemplary for medium (controls, oxylipins result from the FBS), 9(10)-EpOME and 12(13)-EpOME incubations at 50 µM. All data are shown as mean + SD (n=3).

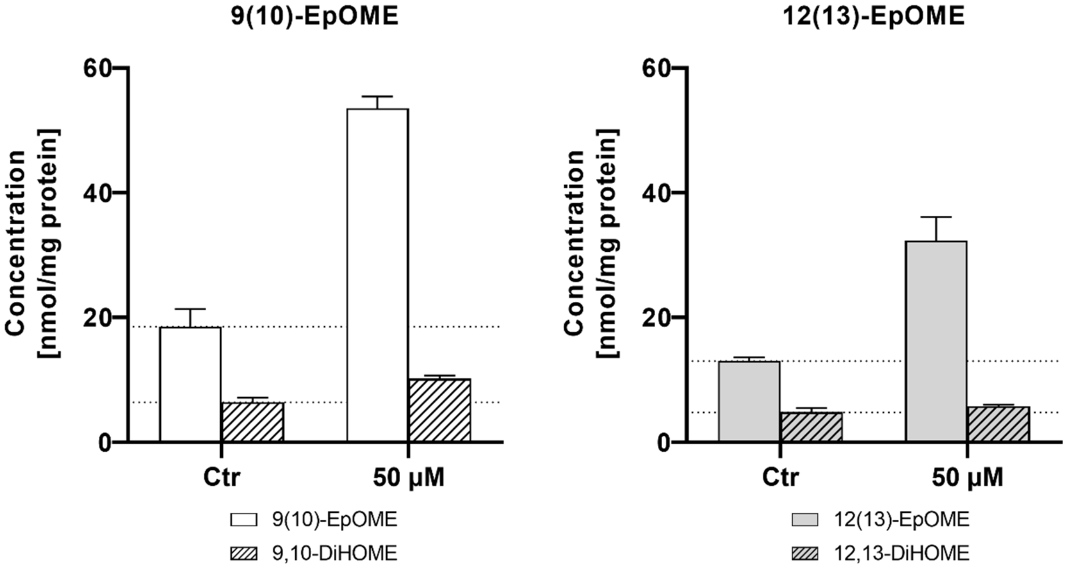

Figure S2: Concentration of total oxylipins in Caco2 cells after incubation with 50 µM epoxy-LA. Cells were incubated with vehicle control (0.1% ethanol *v*/*v*, Ctr), 50 µM of 9(10)-EpOME or 12(13)-EpOME and total oxylipins were determined by LC-ESI(-)-MS/MS after 2 h. All data are shown as mean + SD (n=3).

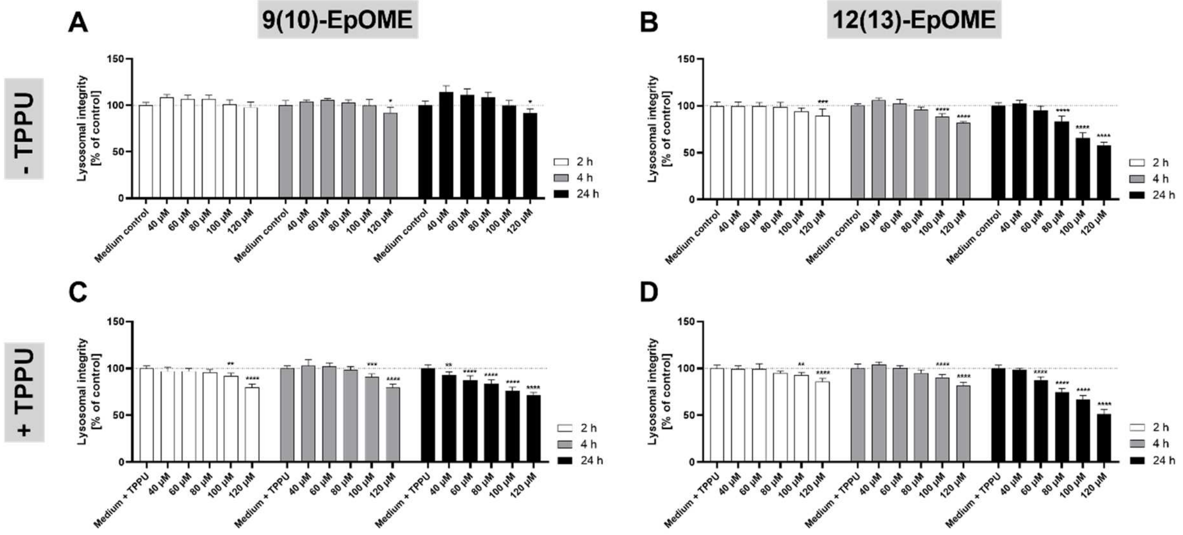

Figure S3: Comparison of cytotoxicity tested by neutral red uptake assay of HepG2 cells of Epoxy-LA with and without sEH inhibition. Cells were incubated with 9(10)-EpOME (A, C) and 12(13)-EpOME (B, D) for 2, 4, and 24 h with (C, D) and without (A, B) 1 µM of sEH inhibitor TPPU [1] and lysosomal integrity was compared to respective medium controls. All data are presented as mean + SD (n=5-6) (Two-

way ANOVA followed by Dunnett’s multiple comparisons test, *p<0.05; **p<0.01; ***p<0.001; ****p<0.0001).

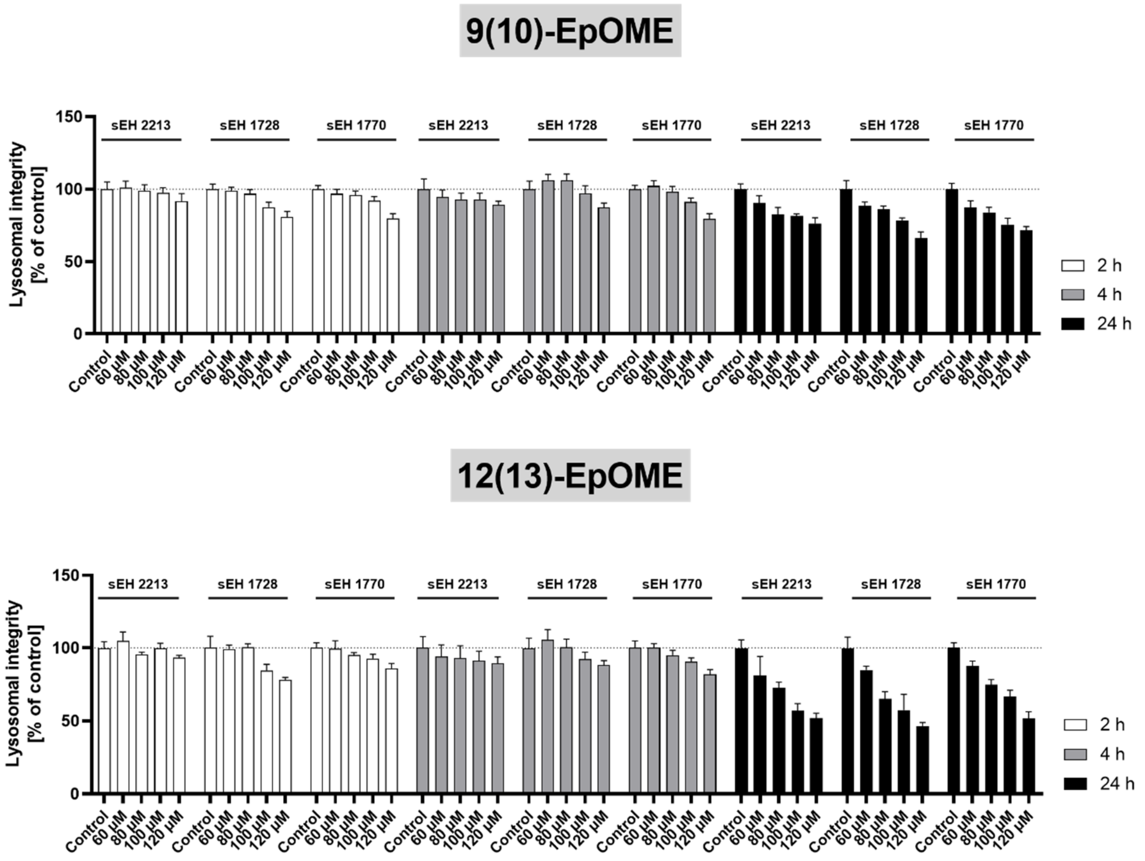

Figure S4: Comparison of cytotoxicity tested by neutral red uptake assay of HepG2 cells of Epoxy-LA with three different sEH inhibitors. Cells were incubated with 9(10)-EpOME and 12(13)-EpOME for 2, 4, and 24 h while co-incubating with the sEH inhibitors 2213 [1], *t*-TUCB (1728) [2] and TPPU (1770) [1] at 1 µM each, and lysosomal integrity was compared to medium controls containing the respective sEH inhibitor. All data are presented as mean + SD (n=6).

References

1. Rose, T. E.; Morisseau, C.; Liu, J. Y.; Inceoglu, B.; Jones, P. D.; Sanborn, J. R.; Hammock, B. D., 1-Aryl-3-(1-acylpiperidin-4-yl)urea inhibitors of human and murine soluble epoxide hydrolase: structure-activity relationships, pharmacokinetics, and reduction of inflammatory pain. *J Med Chem* **2010,** *53* (19), 7067-75 https://doi.org/10.1021/jm100691c.
2. Hwang, S. H.; Tsai, H. J.; Liu, J. Y.; Morisseau, C.; Hammock, B. D., Orally bioavailable potent soluble epoxide hydrolase inhibitors. *J Med Chem* **2007,** *50* (16), 3825-40 https://doi.org/10.1021/jm070270t.
